# EFFECT OF A HEARING INTERVENTION ON THREE-YEAR CHANGE IN BRAIN MORPHOLOGY: ACHIEVE RANDOMIZED TRIAL

**DOI:** 10.1093/geroni/igae098.0055

**Published:** 2024-12-31

**Authors:** James Pike

**Affiliations:** New York University, New York, New York, United States

## Abstract

Prior studies among older adults have documented associations between hearing loss and changes in brain morphology. Whether interventions involving hearing aids can reduce age-related atrophy is unknown. A substudy within the Aging and Cognitive Health Evaluation in Elders (ACHIEVE, Clinicaltrials.gov Identifier: NCT03243422) randomized controlled trial tested the effect of a best-practices hearing intervention versus health education control on three-year change in cortical thickness among older adults with hearing loss. The study enrolled 977 community-dwelling adults aged 70-84 years at baseline (2018-2019) with untreated hearing loss (better ear pure tone average [0.5-4 kHz] ≥30 and <70 dB HL) and without substantial cognitive impairment. Participants were randomized to a hearing intervention (provision of hearing aids and related technologies, counseling, and education) or a health education control (individual sessions with an educator covering health-related topics). Three-dimensional magnetic resonance imaging was performed on 3 Tesla Siemens scanners in a subsample of 445 participants (50.3% women, 11.7% Black) at the baseline and three-year follow-up. Mixed effects models were used in intention-to-treat analyses to estimate three-year change in cortical thickness. Compared to the health education control, the hearing intervention exhibited a nominally protective effect on three-year change in average cortical thickness. The greatest effect size was observed in the occipital lobe, while the smallest effect size was detected in the temporal lobe. Statistically significant effects were detected in the pars orbitalis, rostral anterior cingulate, posterior cingulate, and isthmus cingulate. The results suggest that a hearing intervention may reduce cortical thinning among older adults.

